# Fruit and Vegetable Consumption and Cognitive Disorders in Older Adults: A Meta-Analysis of Observational Studies

**DOI:** 10.3389/fnut.2022.871061

**Published:** 2022-06-20

**Authors:** Yuhan Zhou, Jieyuan Wang, Limin Cao, Mengyuan Shi, Huiyuan Liu, Yuhong Zhao, Yang Xia

**Affiliations:** ^1^Department of Clinical Epidemiology, Shengjing Hospital of China Medical University, Shenyang, China; ^2^Tibet Military Region Centers for Disease Control and Prevention of PLA, Tibet, China; ^3^The Third Central Hospital of Tianjin, Tianjin, China

**Keywords:** fruit, vegetable, cognitive disorders, meta-analysis, dose-response

## Abstract

**Objectives:**

The aim of this meta-analysis was to assess the quantitative associations between fruit and vegetable intake and cognitive disorders in older adults.

**Design:**

A meta-analysis.

**Setting and Participants:**

We used the PubMed, Web of Science and Scopus databases for a literature search to 12 April 2022. We preliminarily retrieved 11,759 studies, 16 of which met the inclusion criteria including six cross-sectional studies, nine cohort studies and one case-control study, incorporating 64,348 participants and 9,879 cases.

**Methods:**

Using the three databases, we identified observational studies exploring the association. The pooled odds ratios (ORs) and 95% confidence intervals (CIs) were calculated using a random effects model.

**Results:**

Sixteen studies were included in the meta-analysis, and the results showed that increased fruit and vegetable consumption in older adults was associated with a decline in the prevalence of cognitive disorders (OR: 0.79, 95% CI: 0.76–0.83). Moreover, intake of fruits (OR: 0.83, 95% CI: 0.77–0.89) and vegetables (OR: 0.75, 95% CI: 0.70–0.80) alone were both associated with a lower prevalence of cognitive disorders. Subgroup analyses indicated that the intake of fruits and vegetables was associated with the prevalence of cognitive impairment (OR: 0.72, 95% CI: 0.76–0.80) and dementia (OR: 0.84, 95% CI: 0.78–0.91) but not Alzheimer’s disease (OR: 0.88, 95% CI: 0.76–1.01).

**Conclusion and Implications:**

Our meta-analysis provides evidence that the intake of fruits and vegetables is inversely proportional and linearly associated with the prevalence of cognitive disorders in older adults. Future research is required to further investigate the preventive effects of the frequency, quantity, and duration of eating vegetables and fruits on cognitive disorders in older adults.

## Introduction

With the rapid development of various countries, population aging is accelerating around the world, especially in developing countries (1). Alongside this, a series of age-related public health problems are growing in prominence, including dementia, mild cognitive impairment (MCI), and Alzheimer’s disease (AD) (2). Age-related cognitive disorders will become more widespread, mainly including delirium, mild cognitive impairment (MCI), and major neurocognitive disorders [such as dementia, Parkinson’s disease (PD), and AD] (3). Among them, MCI is a transitional or a gray area between normal aging and the onset of dementia in which memory, attention, and cognitive function are degraded, but functional independence remains intact (4). The incidence of MCI is 21.5–71.3 per 1,000 person-years for older groups (5). Progression of MCI over 2 years is marked by a gradual decline in intelligence, with personality changes of varying degrees (6). As reported previously, 46.8 million people worldwide suffered from dementia in 2015. By 2050, this figure is estimated to reach up to 131.5 million, increasing the burden on society and families, with AD being the most frequent cause (7). In 2006, the number of patients with AD reached 26.6 million worldwide. It is estimated that this number will reach 106.8 million by 2050 (8). Moreover, the latest research has shown that the prevalence of AD varies greatly worldwide and that the standardized prevalence (all age groups) is between 207 and 812 per 10,000 people according to door-to-door surveys (9). Hence, it is of great clinical and public health significance to find measures to reduce or prevent the incidence of dementia.

The impact of nutritional factors on cognition is very important, as it is a measure that can be changed easily. However, in the context of nutritional epidemiology, the factors that protect against cognitive disorders in older adults are unclear. Previous research has found that nutritional factors play an essential role in brain aging (10). A randomized controlled trial showed that the prevalence of cognitive disorders and related brain diseases can be reduced by consuming specific macronutrients and micronutrients in a balanced diet (11). Certain diets are very important to optimizing cognition and reducing the prevalence of cognitive disorders. For example, the consumption of strawberries, tofu, and edible mushrooms has been found to be associated with the abated prevalence of cognitive disorders (12–14). Overall, these studies demonstrate an association between the intake of fruits and vegetables and a lower prevalence of dementia and cognitive decline. However, published meta-analyses have paid less attention to older adults and have not evaluated the dose-response relationship thoroughly (15–17). Therefore, on this basis, we searched for observational studies published in recent years and carried out a meta-analysis specifically on the older population to assess quantitatively the relationship between fruit and vegetable intake and cognitive disorders.

## Materials and Methods

### Literature Search Strategy

We complied strictly with the Guidelines for Meta-Analyses and Systematic Reviews of Observational Studies when conducting the meta-analysis (18). Two researchers conducted a systematic literature search of the Web of Science, PubMed, and Scopus electronic databases (1970.01-2022.04) to find appropriate articles. Electronic searches included MeSH and free text searches. The following key words were used: (“vegetable” OR “vegetables” OR “fruit” OR “fruits” OR “vegetable products” OR “dried fruit”) combined with (“Alzheimer’s disease” OR “cognitive impairment” OR “Alzheimer’s type” OR “cognitive decline” OR “dementia” OR “MCI” OR “mild neurocognitive disorder” OR “mild cognitive impairment” OR “cognitive impair*” OR “memory impair*” OR “Alzheimer’s disease” OR “dementia*” OR “cognitive disorders” OR “cognitive disorder*” OR “cognitive defect” OR “memory disorder*” OR “AD” OR “executive function” OR “Alzheimer*”). Manuscripts published in languages other than English were excluded. In addition, we searched manually for a list of potential and related articles.

### Study Selection

The inclusion criteria for the selection of studies were as follows: (1) the manuscript reported an observational study; (2) the outcome of the study was associated with the occurrence of cognitive disorders; (3) the variables that explored in the study population included dietary intake of fruits and/or vegetables; (4) studies indicated an relationship between vegetable and fruit intake, and the association strength index odds ratio [(OR)/hazard ratio (HR)/relative risk (RR)] with cognitive disorders and the 95% confidence interval (CI) were included; and (5) participants in the original study were aged over 60 years. The exclusion criteria for the study were as follows: (1) research data included only nutrients within fruits and vegetables, such as protein, vitamins or fiber; or (2) the exposure variable was the intake of specific types of vegetables or fruits.

### Data Extraction and Quality Assessment

A research assistant collected the following data from the literature: first author, type of study design, country, sample size, age, follow-up time, disease type (cognitive impairment, AD and dementia), disease determination, exposure variables (fruits and/or vegetables), number of cases, lowest and highest categories of fruit and/or vegetable intake, exposure assessment, risk estimates with CIs, and confounders. Disagreement during the analysis was solved through discussion with the third author. The Newcastle-Ottawa Scale (19) and the Agency for Healthcare Research and Quality (20) were used for quality assessment.

### Statistical Analysis

We conducted a meta-analysis of risk assessments for cognitive disorders; the highest and lowest categories of fruit and vegetable intake were compared. In a meta-analysis, we assumed that RRs and HRs were approximately equivalent to ORs when the prevalence of the outcome was low (21). Given the limited amount of research on each disease, we collected data associated with cognitive impairment, AD and dementia. Subsequently, we used a generic inverse-variance method (random-effects model) (22) to calculate the total association strength index. We used an Excel Macro file (23) to convert the effect quantity and 95% CI referring to groups other than the lowest dose group in the original study into the effect quantity and 95% CI referring to the lowest dose group, because these two types of studies cannot be directly combined. Moreover, we carried out a dose-response meta-analysis based on the daily intake of vegetables and fruits. Dose-response categorical meta-analyses require data for the distribution of exposures across ≥3 categories, and only four studies met this condition (24–27).

He et al. assessed the average weight of a series of common fruits and vegetables, using serving size weights for a 0.5 cup standard serving (28); it is estimated that the average daily intake of fruits is 80 g and that of vegetables is 77 g. According to a previous study (15), to obtain a better fit in the dose-effect model, for studies that reported results for daily consumption of fruit and/or vegetable in servings only, we derived grams by assuming that the average daily serving is 80 g for fruits and 77 g for vegetables. In the dose-response meta-analysis, the model was fitted in increments of 80 g of fruits, 77 g of vegetables, and 78.5 g of fruits and vegetables combined per day. We also conducted a sensitivity analysis to remove each individual study from the meta-analysis to determine the impact of each study on the overall results. Funnel plots, followed by Egger’s and Begg’s tests, were used to estimate publication bias. In addition, we carried out subgroup analyses using pre-specified characteristics (country, gender, exposure assessment method, disease type, design type, number of cases, and type of exposure). The statistical analysis was carried out using Stata version 17.0 software (Stata Corp., College Station, TX, United States).

## Results

### Search Result

**FIGURE 1:**
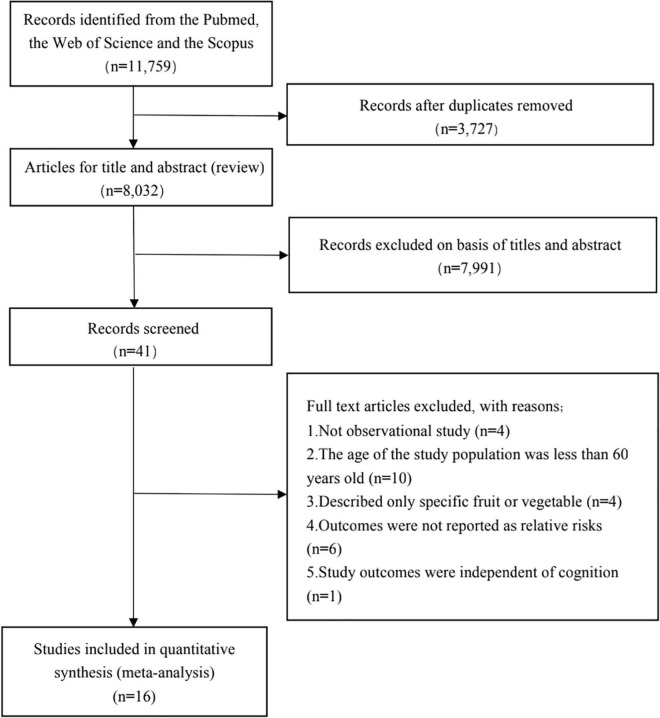
Flowchart of study selection about the relationship between fruits and vegetables intake and cognitive disorders.

Figure 1 presents the selection flow chart for this study. Briefly, 11,759 articles were identified in the PubMed, Web of Science and Scopus databases. Ultimately, 16 studies (including six cross-sectional studies, nine cohort studies, and one case-control study) conformed to the inclusion criteria and were included in the meta-analysis. A total of 64,348 participants and 9,879 patients with cognitive disorders, AD, or dementia were included, with an average follow-up time of 9.8 years. Table 1 describes the characteristics of each study. We recorded the different consumption frequencies of vegetables and fruits and the RR/HR/OR of cognitive disorders in accordance with the highest and lowest classification of fruit and vegetable intake. Supplementary Tables 1, 2 indicate the quality of the estimated results of the included studies. In accordance with the quality estimated by the Newcastle-Ottawa scale, the mean value for the one case-control and nine cohort studies was 7.6 stars. We considered a study awarded 5–9 stars to be of medium- or high-quality, because the criteria for medium or high quality have not been established. All documents were of medium or high quality, with scores of 5–9. The Agency for Healthcare Research and Quality scale was used to evaluate the cross-sectional studies, including 11 items. Each item was rated as “yes” (1 point), “no” or “unclear” (0 points); 0–3 points indicated low-quality literature, 4–7 points medium-quality literature, and 8–11 points high-quality literature. All documents were of medium or high quality, with scores of 6–8.

**TABLE 1:** Characteristics of the included studies in this meta-analysis.

References	Country	Study design	Sample size	Age	Disease assessment	Number of cases	Disease type	Exposure assessment	OR/HR (95% CI)	Adjusted covariates
Dai (60)	America	Cohort	1,836	≥65	NINCDS-ADRDA	81	AD	FFQ	^a^V + F: 0.24 (0.09-0.61)	Age, education, years of education, dietary intake of vitamins C, E, and β-carotene
Barberger-Gateau (61)	France	Cohort	8,085	≥65	Neurological exam, DSM-IV and NINCDS-ADRDA	281	Dementia	FFQ	^a^V + F:0.72 (0.53-0.97)	Age, gender, education, city, income, marital status, ApoE genotype, BMI, and diabetes
Vercambre (62)	France	Cohort	4,809	76–82	Observed Cognitive Deterioration Scale	598	Recent cognitive impairment	DHQ	^a^V: 0.80 (0.65-0.98) V + F:0.86 (0.70–1.06)	Age, education level, BMI, physical activity, daily energy intake, smoking, supplement of vitamin D and/or Ca, supplement of other vitamins or minerals, use of postmenopausal hormones, history of depression, history of cancer, history of CHD, history of stroke, history of diabetes mellitus, history of hypertension, and history of hypercholesterolaemia
Roberts et al. (27)	America	Cross-sectional	1,233	70–89	CDR	163	MCI	FFQ	^a^V: 0.66 (0.44-0.99) F: 0.92 (0.61-1.38)	Age, gender, years of education, total energy intake, ApoE ε4, stroke, coronary heart disease, depressive symptoms
Ritchie (63)	France	Cohort study	1,433	≥65	Standardized interview incorporating cognitive testing	405	MCI or Dementia	Nutritional questionnaire	*^b^*V + F:1.26 (1.02-1.56)	Age and gender
Lee et al. (26)	Hong Kong, China	Cross-sectional	285	≥60	DSM-VI and CDR	146	Dementia	MNA	^a^V + F: 0.26 (0.07-0.97)	Age, gender, and education
Wu et al. (24)	Tai Wan, China	Cross-sectional	2,119	≥65	MMSE	472	Cognitive impairment	Questionnaire for lifestyle	^a^V + F: 0.81 (0.49-1.33)	Age, gender, educational level, marital status, social support, hyperlipidemia, stroke, physical function, depressive symptoms, self-rated health, cigarette smoking, physical activity, coffee intake, tea intake, multivitamin intake, and BMI
Chen (64)	China	Case-control	5,691	≥65	MMSE	1,306	Cognitive impairment	FFQ	^a^V: 0.66 (0.58-0.75) F: 0.76 (0.60–0.97)	Age, gender, marital status, financial status, residential area, BMI, hypertension, diabetes, smoking, alcohol, tea drinking, and exercise habits
Chan (65)	Hong Kong, China	Cross-sectional	3,670	≥65	CSI-D	877	Cognitive impairment	FFQ	^a^V + F: men: 1.09 (0.72-1.67) women: 0.73 (0.54-1.00)	Age, BMI, PASE, energy intake, educational level, Hong Kong ladder, community ladder, smoking status, alcohol use, No. of ADLs, GDS y of DM, category, self-reported history hypertension, and CVD/stroke
Pastor-Valero et al. (25)	Brazilian	Cross-sectional	1,849	≥65	CSI-D	147	Cognitive impairment	FFQ	^a^V + F:0.53 (0.31-0.89)	Age and gender
Lee (66)	China	Cohort	17,700	≥65	ICD-10 and CDR	1,620	Dementia	FFQ	^a^V: 0.88 (0.73-1.06) F: 0.86 (0.74-0.99) V + F:0.75 (0.60-0.95)	Age, gender, education, major chronic diseases, physical exercise and smoking
Kimura (67)	Japan	Cohort	1,071	≥60	DSM-III-R and NINDS-AIREN	430	Dementia and AD	FFQ	^a^Dementia V: 0.73 (0.56-0.96) F: 0.90 (0.69-1.17) AD V: 0.69 (0.49-0.98) F: 1.03 (0.74-1.44)	age, gender, educational level, history of stroke, diabetes, systolic blood pressure, use of anti-hypertensive agents, electrocardiogram abnormalities, serum total cholesterol, BMI, current drinking, current smoking, regular exercise, and intakes of total energy, protein, fat, and carbohydrate
Fischer (68)	Germany	Cohort	2,622	≥75	DSM-IV, ICD-10 and NINDS-AIREN	418	Dementia and AD	“Cognitive health” food intake screener	^a^V + F:1.08 (0.80-1.46)	Age, gender, BMI, education, and APOEε4 status
An (69)	China	Cohort	4,749	≥80	MMSE	1,958	Cognitive impairment	Self-reported information on dietary intake	^a^V: 0.75 (0.64-0.87) F: 0.79 (0.70-0.90)	Age, gender, education level, living arrangement, place of residence, body weight, smoking status, alcohol consumption status, exercise status, self-rated health, chronic disease, the Katz activities of daily living limitation
Ngabirano (70)	France	Cohort	5,934	≥65	DSM-VI	662	Dementia and AD	FFQ	*^b^*Dementia V: 1.18 (0.92-1.51) F: 1.03 (0.75-1.42) V + F:0.68 (0.36-1.29) AD V: 1.17 (0.88-1.56) F: 1.11 (0.77-1.60) V + F: 0.79 (0.39-1.63)	Inclusion center, gender, marital status, income, level of education, APOE 4, smoking, alcohol consumption, physical activity frequency and energy intake
Xu (71)	China	Cross-sectional	1,262	≥65	MMSE	315	MCI	FFQ	^a^F: 0.49 (0.35-0.69)	Age, gender, education, marital status, smoking, alcohol drinking, energy intake, diabetes mellitus, hypertension, physical activity, MNA-SF and IADL scores

*AD, Alzheimer’s disease; BMI, body mass index; CDR, Clinical Dementia Rating Scale; CI, confidence interval; CHD, coronary heart diseases; CSI-D, Community Screening Instrument for Dementia; DSM-IV, Diagnostic and Statistical Manual of Mental Disorders, Fourth Edition; F, fruit; F + V, fruit and vegetable; FFQ, food frequency questionnaire; HR, hazard ratio; IADL, Instrumental Activities of Daily Living scale; ICD-10, the 10th revision of the International Statistical Classification of Diseases and Related Health Problems; MMSE: Mini-Mental State Examination; MNMA-SF, Mini Nutritional Assessment-Short Form; MoCA, Montreal Cognitive Assessment; NINCDS-ADRDA, National Institute of Neurological and Communicative Diseases and Stroke-Alzheimer’s Disease and Related Disorders Association; NINCDS-AIREN, the National Institute of Neurological and Communicative Disorders and Stroke and the Alzheimer’s Disease and Related Disorders Association; OR, odds ratio; PASE, Physical activity Scale for the Elderly; V, vegetable.*

*^a^Compared with the lowest category of the study, the highest category of cognitive impairment risk.*

*^b^Compared with the highest category of the study, the lowest category of cognitive impairment risk.*

### Association Between Fruit and Vegetable Intake and Cognitive Disorders

**FIGURE 2:**
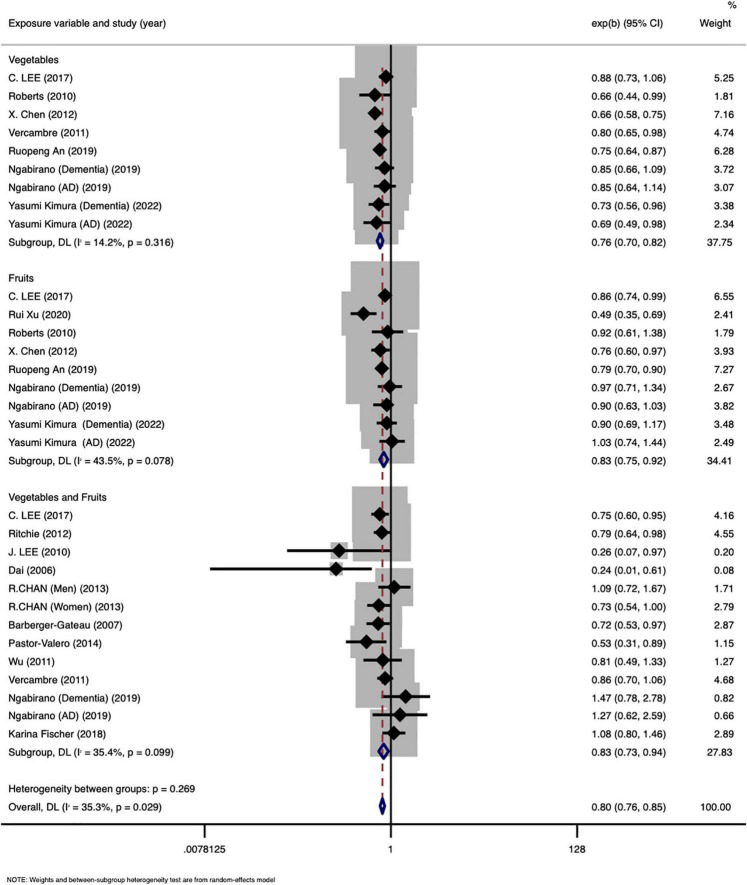
Meta-analysis of the association between fruits and vegetables consumption and the prevalence of cognitive disorders.

A total of 16 articles, including 31 effect groups, reported the relationship between fruit and vegetable intake and cognitive disorders in the older age group. The results of the meta-analysis of ORs in accordance with the highest and lowest categories of fruit and vegetable intake are shown in Figure 2. The pooled results demonstrated that a high intake of fruits and vegetables was associated with a declined in the prevalence of cognitive disorders (OR: 0.82, 95% CI: 0.75–0.90). Intake of fruits (OR: 0.83, 95% CI: 0.77–0.89) or vegetables (OR: 0.75, 95% CI: 0.70–0.80) separately was also associated with a reduced prevalence of disease in older adults. Remarkable heterogeneity was found in the pooled analysis (*I*^2^ = 35.3%, *P* < 0.05).

### Subgroup Analysis and Sensitivity Analysis

The subgroup analyses (Table 2) showed that higher fruit and vegetable intake was negatively associated with the prevalence of cognitive disorders (OR: 0.76, 95% CI: 0.72–0.81) both in the Chinese population (OR: 0.76, 95% CI: 0.72–0.81), and other countries (OR: 0.84, 95% CI: 0.79–0.90). A gender-based subgroup analysis demonstrated that, in women (OR: 0.73, 95% CI: 0.54–0.99) and both men and women participants combined (OR: 0.79, 95% CI: 0.76–0.83), higher fruit and vegetable intake was associated with a lower prevalence of cognitive disorders when compared with lower intake. However, no significant association was found in the subgroups of men (OR: 1.09, 95% CI: 0.72–1.66). In the subgroup analysis of disease types, the intake of vegetables and fruits was significantly association with prevalence of cognitive impairment (OR: 0.76, 95% CI: 0.72–0.80) and dementia (OR: 0.84, 95% CI: 0.78–0.91), but there was no association with AD (OR: 0.88, 95% CI: 0.76–1.01). According to the type of study design, we divided all studies into three subgroups. However, all three subgroups, cohort studies (OR: 0.83, 95% CI: 0.79–0.87), cross-sectional studies (OR: 0.70, 95% CI: 0.61–0.82), and case-control studies (OR: 0.68, 95% CI: 0.61–0.76), indicated that intake of vegetables and fruits was negatively associated with the presence of cognitive disorders. A subgroup analysis based on exposure assessment methods showed that there was a negative association between vegetable and fruit intake and cognitive disorders as measured by the Food Frequency Questionnaire (OR: 0.79, 95% CI: 0.75–0.83) and other assessment methods (OR: 0.80, 95% CI: 0.75–0.86). In the subgroup analysis based on the number of cases, no remarkable heterogeneity was found among the subgroups. In the sensitivity analysis (Supplementary Figure 1), the comprehensive results did not change after excluding each article successively.

**TABLE 2:** Subgroup analyses of relationship between fruits and vegetables intake and cognitive disorders.

Subgroup	Number of effects	Effect size (95% CI)	*I*^2^ (%)
**Country**			
China	12	0.76 (0.72–0.81)	50.0%
Others	19	0.84 (0.79–0.90)	46.1%
**Gender**			
Men	1	1.09 (0.72–1.66)	–
Women	1	0.73 (0.54–0.99)	–
Both	29	0.79 (0.76–0.83)	36.2%
**Disease type**			
Cognitive impairment	15	0.76 (0.72–0.80)	42.8%
Dementia	10	0.84 (0.78–0.91)	12.7%
Alzheimer’s Disease	6	0.88 (0.76–1.01)	5.80%
**Study design**			
Cohort	21	0.83 (0.79–0.87)	0.00%
Cross-sectional	8	0.70 (0.61–0.82)	49.5%
Case-control	2	0.68 (0.61–0.76)	2.90%
**Exposure assessment**			
FFQ	23	0.79 (0.75–0.83)	42.9%
Others	8	0.80 (0.75–0.86)	9.4%
**Number of cases**			
≥200	26	0.80 (0.76–0.83)	35.7%
<200	5	0.68 (0.53–0.87)	32.3%

*CI, confidence interval; FFQ, food frequency questionnaire.*

### Dose-Response Association Analysis

**FIGURE 3:**
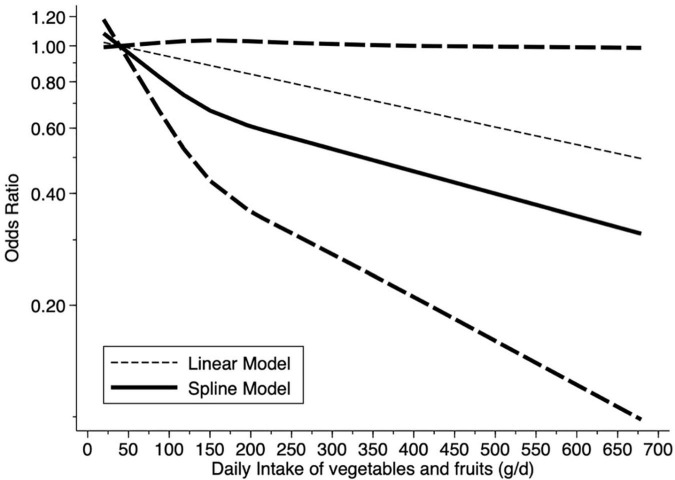
Dose-response relationship between fruits and vegetables intake and prevalence of cognitive disorders.

Dose-response meta-analysis requires the inclusion of data for at least three exposure categories, and therefore only five estimates from four separate studies were analyzed. As shown in Figure 3, in older adults there was an obvious linear dose-response relationship between the intake of vegetables and fruits and the prevalence of cognitive disorders (*P* = 0.03).

### Publication Bias

The funnel plot (Supplementary Figure 2) indicated that, in the meta-analysis and dose-response meta-analysis, the comprehensive effect size distribution of the study was relatively symmetrical, suggesting that there was no publication bias. Begg’s regression test also showed no potential publication bias in the meta-analysis (*P* = 0.51) and dose-response meta-analysis (*P* = 0.22).

## Discussion

A total of 16 articles, including 31 effect groups, was used to explore the relationship between fruit and vegetable intake and cognitive disorders in older adults. Following the data analysis, we found that consumption of fruits and/or vegetables was associated with a decline in the prevalence of cognitive disorders. The results of the dose-response relationship indicated that an increase in the daily intake of fruits and/or vegetables was significantly associated with a reduction of cognitive impairment in older adults.

Several previous meta-analyses (15–17) have studied the impact of vegetable and fruit consumption on cognition among adults and found a negative association, which is consistent with our findings. Considering that cognitive decline is an age-related disease, we investigated its relationship with fruit and vegetable consumption among older adults. Consistently, consumption of fruits and/or vegetables showed a dose-response relationship with reduced prevalence of cognitive disorders. Furthermore, published meta-analyses have only reported that the combination of fruits and vegetables showed a beneficial effect on cognitive impairment and dementia. In addition to this, we found that fruit or vegetable intake alone was also negatively associated with cognitive disorders.

The beneficial effect of a diet containing a high proportion in fruits and vegetables on cognition in older adults is perhaps due to the rich supply of antioxidant nutrients (29, 30). Antioxidants may benefit cognitive function by influencing various metabolic pathways. In addition to participating in AD pathology, oxidative stress is also considered to be a crucial component of many steps in the progression of vascular cognitive impairment and dementia (31). In numerous studies on the abundance of antioxidant nutrients in fruits and vegetables (32–36), the results showed that older adults who took in more nutrients as food supplements or had higher concentrations in plasma had better cognitive status. Some epidemiological studies have reported that a greater intake of antioxidants can reduce cognitive disorders or dementia (37, 38). The consumption of specific kinds of fruits and vegetables, such as berries, soybeans and bean products, dark green leafy vegetables, nuts, cruciferous vegetables, root vegetables, fungi, and nuts, may also protect against the risk of cognitive disorders (12–14, 32–35, 39); however, because of limited data, these studies were not included in our meta-analysis. In addition, a previous study (40) indicated that nutrition regulates the immune system and changes the neuroinflammatory process associated with the pathogenesis of AD and the progression of neurodegeneration. Thus, the anti-inflammatory properties of fruits and vegetables may be another beneficial factor for cognitive function in older adults. In two cohort studies (41, 42), researchers investigated inflammation-related dietary patterns. Generally, an anti-inflammatory diet includes mainly vegetables, fruits, whole beans, and seafood. These studies found that more inflammatory diet patterns are associated with an accelerated decline in cognitive ability. Compared with the lowest quartile, overall cognitive and reasoning ability declined the most in those in the highest quartile, who consumed the most inflammatory diet. Moreover, in a recent review, researchers found a beneficial effect of an anti-inflammatory diet on cognitive health results in older adults (43).

Both stroke and AD have major socio-economic effects, and these two diseases can occur simultaneously and affect each other. The vascular factors predisposing people to cerebrovascular diseases are often associated with the occurrence of AD. Acute stroke is known to raise the risk of cognitive disorders in the older age group (44). A meta-analysis indicated that the intake of fruits and vegetables can reduce the incidence of stroke, which can partially explain its beneficial effect on the vascular components of dementia (45). Fruits and vegetables include high levels of polyphenols and phytoestrogens, which may also play a positive role in reducing the prevalence of cognitive disorders (46). The result of a cohort study in Spain indicated that the intake of lignans and stilbene could improve cognitive function in older adults (47). Some researchers have also found that the more fruit consumed, the better the cognitive performance of older adults (48). However, one cohort study found that eating more vegetables can reduce dementia or cognitive decline, but this was not true of fruits (36). Other studies have shown that different types of vegetables and fruits may have different effects on cognition, depending on the type of cognitive function assessed (such as verbal memory and executive function) (49, 50). Therefore, more studies are required to further evaluate the effect of fruits and vegetables individually on the risk of developing cognitive disorders.

Our country-based subgroup analysis showed that higher fruit and vegetable intake was negatively associated with the prevalence of cognitive impairment in the Chinese population and other countries, and it is worth noting that this negative association was more pronounced in the Chinese population. We speculated that this difference may be attributed to diverse dietary patterns and cooking methods. Specifically, the traditional Chinese diet pattern is characterized by whole grains, tea, soybeans, and vegetables (51), as well as a high content of phytochemicals (catechol and caffeine). In contrast, the Western diet, marked by the intake of large amounts of high-fat dairy products, meat, butter, eggs, and refined sugar, is associated with a greater decline in cognitive ability (52). In another study of the older adults in China (53), maintaining healthy eating habits reduced the risk of developing cognitive disorders. As a potential strategy to reduce brain diseases, a healthy diet has attracted increased attention in the scientific community recently. Many studies (54–56) have shown that there is an association between dietary patterns and cognitive function. Healthy dietary patterns are mostly based on eating rice and flour, red meat, seafood, vegetables, and fruits, which can prevent cognitive disorders (54). Among modern residents of New York, adherence to the Mediterranean diet has been associated with a slower decline in cognitive ability and a lower risk of AD (57), as measured by global scores, as well as higher language learning and memory abilities (58). This may partially explain the more pronounced negative association between the consumption of vegetables and fruits and the risk of cognitive decline in Chinese population.

The present study has some advantages over previous reports. First, our meta-analysis included numerous people with long-term follow-up in a few prospective cohort studies. Second, we evaluated the dose-response relationship and detected a linear association based on the intake of vegetables and fruits. Given the high incidence of cognitive disorders in older adults, this study will support the development of dietary guidelines for older adults and help to develop preventive interventions aimed at reducing the prevalence of dementia-related diseases. Finally, our results showed that eating vegetables alone was associated with a reduced prevalence of cognitive disorders, which was not found in previous meta-analyses. In addition, to detect potential sources of heterogeneity, we carried out subgroup analyses, used sensitivity analysis to check the robustness of the results, and measured publication bias. The limitations of this study were, first, that the evaluation of fruit and vegetable intake in the evaluated studies was mostly based on self-reported habits; as a result, the data were prone to recall bias. Second, in the quantitative response analysis, only four studies reported the consumption of more than three kinds of fruits and vegetables, so it may have lacked sufficient effectiveness in quantifying the relevant dose-response model. Third, a previous study (59) suggested that using the OR as an approximation of the RR produces progressively larger errors as the outcome rate rises above 1%, therefore, we treated HR/RR as equal to OR, which may have affected the results. Fourth, a meta-analysis cannot solve possible problems with inherent confounding in the included studies; however, most of the studies included had adjusted for the main confounding factors to reduce the potential bias caused by diet and lifestyle factors. Finally, no casual relationships could be established due to the observational nature of the studies. Future randomized controlled trials are necessary to assess the casual relationships. Following cognitive impairment, the incidence of AD and dementia rise sharply with age, and the positive effect of fruits and vegetables on cognition is more prominent among older adults. Therefore, further studies are warranted, which involve high-quality research with standardized measurement units, to investigate the potential dose-response effects of different kinds of fruits and vegetables on the risk of developing cognitive disorders and to determine whether the frequency, amount, and duration of vegetable and fruit consumption can effectively improve cognition or prevent the occurrence of cognitive disorders.

## Conclusion and Implications

The results of the current meta-analysis of 16 studies showed that higher fruit and vegetable intake was negatively associated with the prevalence of cognitive disorders in older adults. It will be necessary to conduct further prospective studies to analyze the effects of different varieties of vegetables and fruits on cognitive disorders and accurately quantify the potential dose-response patterns of fruit and/or vegetable consumption.

## Author Contributions

YZho: conceptualization, formal analysis, visualization, and writing – original draft. JW: writing – review and editing and supervision. LC, MS, HL, and YZha: writing – review and editing. YX: conceptualization, resources, writing – review and editing, supervision, and funding acquisition. All authors contributed to the article and approved the submitted version.

## Conflict of Interest

The authors declare that the research was conducted in the absence of any commercial or financial relationships that could be construed as a potential conflict of interest.

## Publisher’s Note

All claims expressed in this article are solely those of the authors and do not necessarily represent those of their affiliated organizations, or those of the publisher, the editors and the reviewers. Any product that may be evaluated in this article, or claim that may be made by its manufacturer, is not guaranteed or endorsed by the publisher.
