# Supplementary material for: Fruit and Vegetable Consumption and Cognitive Disorders in Older Adults: A Meta-Analysis of Observational Studies
Source: Front Nutr. 2022 Jun 20;9:871061. doi: 10.3389/fnut.2022.871061 (PMC9251442; doi:10.3389/fnut.2022.871061)
Supplement: Supplementary file 1 [file Data_Sheet_1.docx]

**Table S1.** **assessment scale of cohort study or case-control study^a^**

| **First author, published year** | **Selection** | **Comparability** | **Outcome** | **Total** |
| --- | --- | --- | --- | --- |
| **Dai (2006)** | 4 | 2 | 3 | 9 |
| **Barberger-Gateau (2007)** | 4 | 2 | 2 | 8 |
| **Vercambre (2009)** | 3 | 1 | 3 | 7 |
| **Ritchie (2010)** | 3 | 1 | 3 | 7 |
| **X.Chen (2012)** | 3 | 1 | 1 | 5 |
| **C. LEE (2017)** | 3 | 2 | 3 | 8 |
| **Karina Fischer (2018)** | 3 | 2 | 3 | 8 |
| **Ruopeng An (2019)** | 4 | 1 | 3 | 8 |
| **Ngabirano (2019)** | 4 | 2 | 2 | 7 |
| **Yasumi Kimura (2022)** | 4 | 2 | 3 | 9 |

^a^The Newcastle–Ottawa Scale was used to assess the quality of studies

**Table S2. Cross-sectional study quality assessment scale^a^**

| **First author, published year** | **Total** |
| --- | --- |
| **Pastor-Gateau (2007)** | 6 |
| **Roberts (2010)** | 8 |
| **J.LEE (2010)** | 6 |
| **Wu (2011)** | 8 |
| **R.CHAN (2013)** | 7 |
| **Rui Xu (2020)** | 8 |

^a^The Agency for Healthcare Research and Quality was used to assess the quality of studies


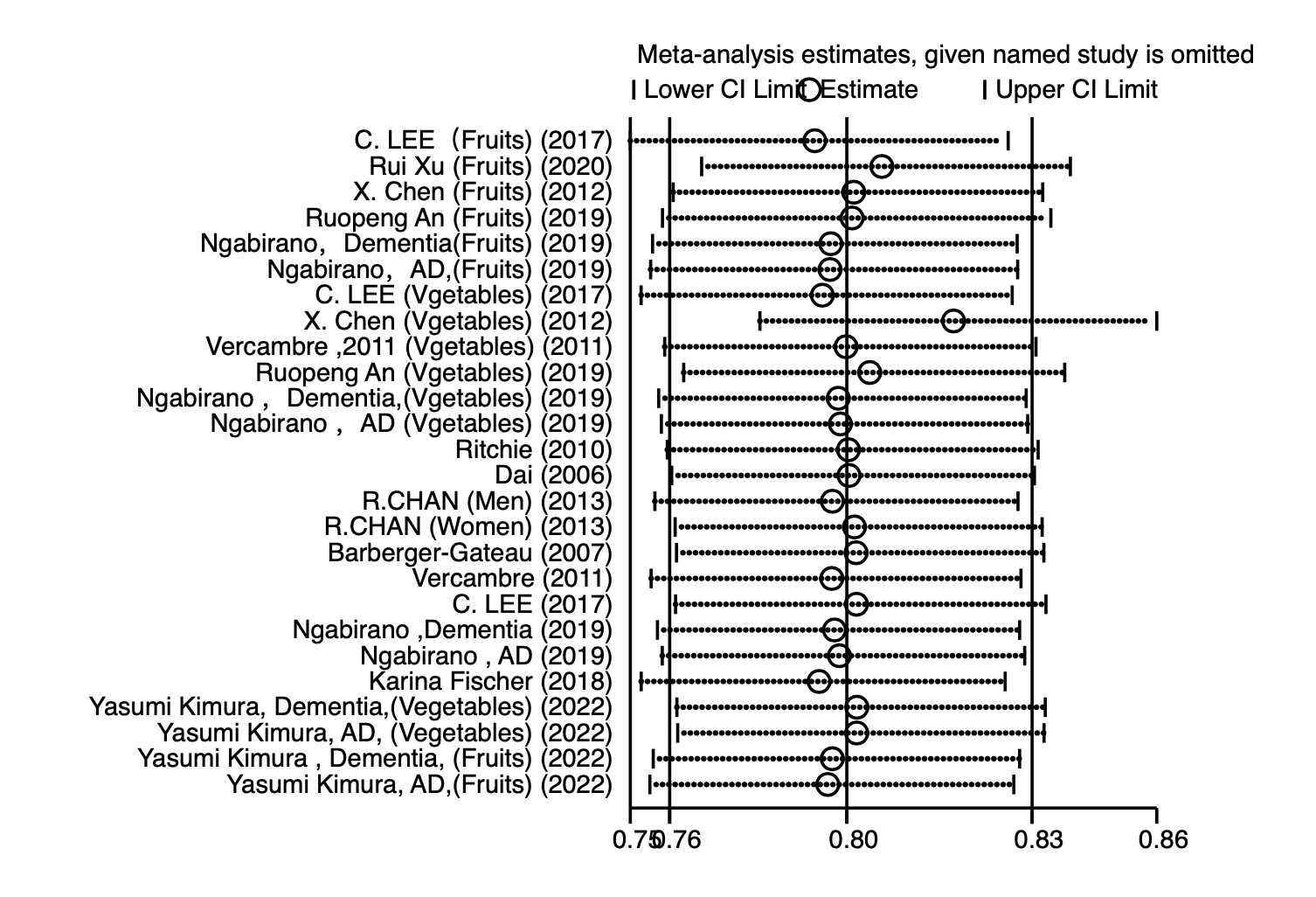


**Figure S1.** **Sensitivity analysis of association between fruits and vegetables intake and the cognitive disorders.**


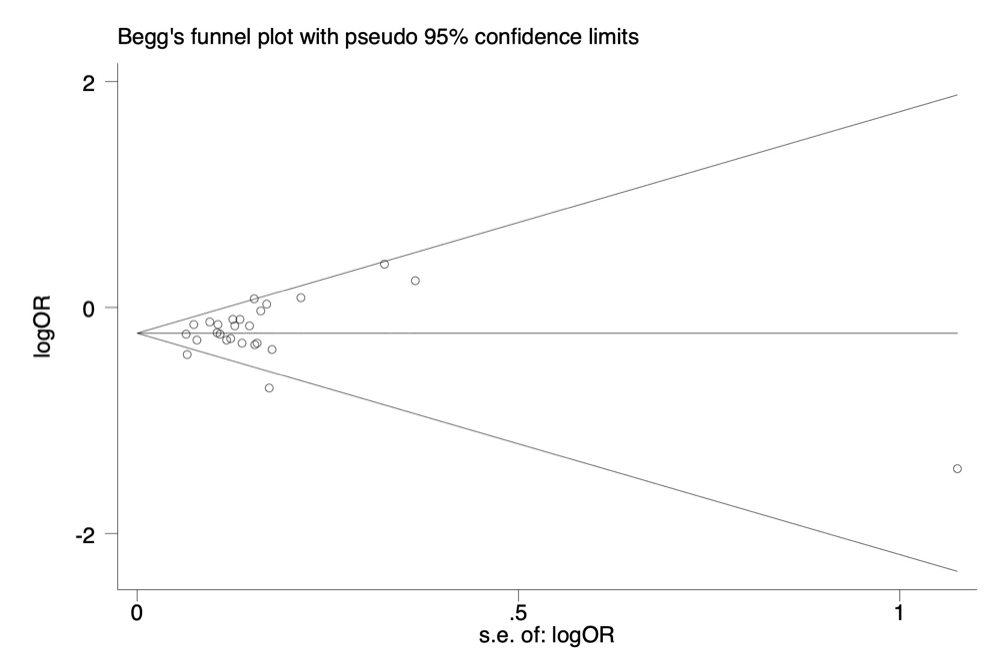


**A: Highest VS. lowest category of fruits and vegetables intake**


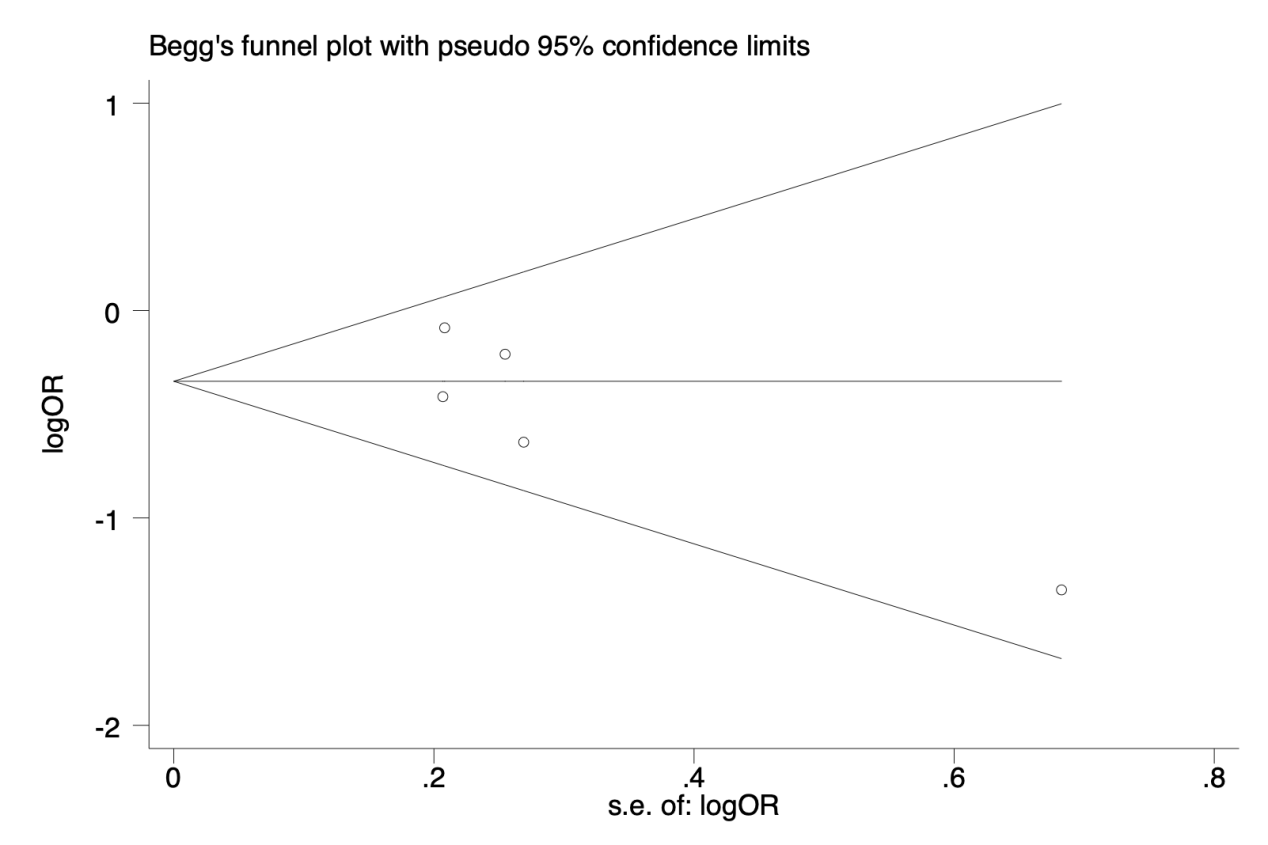


**B: Dose-response meta-analysis**

**Figure S2. Begg’s funnel plot and Egger’s test for identifying publication bias.**
